# 4-Hy­droxy­benzamide 1,4-dioxane hemisolvate

**DOI:** 10.1107/S160053681203437X

**Published:** 2012-08-08

**Authors:** Srinu Tothadi, Gautam R. Desiraju

**Affiliations:** a Solid State and Structural Chemistry Unit, Indian Institute of Science, Bangalore 560 012, India

## Abstract

The asymmetric unit of the title compound, C_7_H_7_NO_2_·0.5C_4_H_8_O_2_, is composed of one 4-hy­droxy­benzamide mol­ecule and half of a 1,4-dioxane mol­ecule. The complete dioxin molecule is generated by crystallographic inversion symmetry. The crystal has an extensive system of hydrogen bonds, in which the three donor H atoms are fully utilized: these result in amide–amide homodimers, and N—H⋯O(dioxane) and O—H⋯O(amide) links.

## Related literature

For the structure and properties of 4-hy­droxy­benzamide and its hydrate, see: Kashino *et al.* (1991 ▶); Perlovich *et al.* (2007 ▶); Hansen *et al.* (2007 ▶).
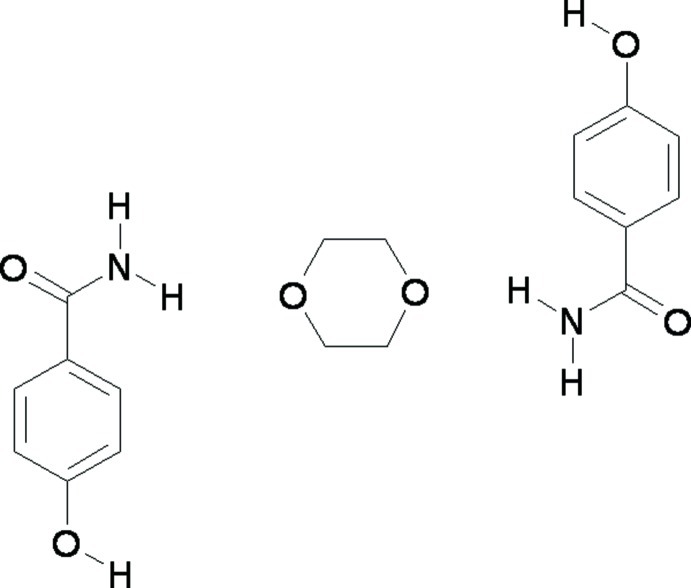


## Experimental

### 

#### Crystal data

C_7_H_7_NO_2_·0.5C_4_H_8_O_2_
*M* *_r_* = 181.19
Monoclinic, 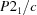

*a* = 5.4062 (15) Å
*b* = 14.530 (3) Å
*c* = 12.027 (2) Å
β = 113.117 (10)°
*V* = 868.9 (3) Å^3^
*Z* = 4
Mo *K*α radiation
μ = 0.11 mm^−1^
*T* = 150 K
0.30 × 0.30 × 0.20 mm

#### Data collection

Rigaku Mercury375R (2x2 bin mode) diffractometer
Absorption correction: multi-scan (*REQAB*; Jacobson, 1998 ▶) *T*_min_ = 0.969, *T*_max_ = 0.979
9077 measured reflections
1987 independent reflections
1841 reflections with *I* > 2σ(*I*)
*R*_int_ = 0.064

#### Refinement

*R*[*F* ^2^ > 2σ(*F* ^2^)] = 0.039
*wR*(*F* ^2^) = 0.111
*S* = 1.01
1987 reflections
162 parameters
All H-atom parameters refined
Δρ_max_ = 0.28 e Å^−3^
Δρ_min_ = −0.22 e Å^−3^

### 

Data collection: *CrystalClear-SM Expert* (Rigaku, 2009 ▶); cell refinement: *CrystalClear-SM Expert*; data reduction: *CrystalClear-SM Expert*; program(s) used to solve structure: *SHELXS97* (Sheldrick, 2008 ▶); program(s) used to refine structure: *SHELXL97* (Sheldrick, 2008 ▶); molecular graphics: *ORTEP-3* (Farrugia, 1999 ▶); software used to prepare material for publication: *PLATON* (Spek, 2009 ▶).

## Supplementary Material

Crystal structure: contains datablock(s) global, I. DOI: 10.1107/S160053681203437X/fy2064sup1.cif

Structure factors: contains datablock(s) I. DOI: 10.1107/S160053681203437X/fy2064Isup2.hkl

Supplementary material file. DOI: 10.1107/S160053681203437X/fy2064Isup3.cdx

Supplementary material file. DOI: 10.1107/S160053681203437X/fy2064Isup4.cml

Additional supplementary materials:  crystallographic information; 3D view; checkCIF report

## Figures and Tables

**Table 1:** Hydrogen-bond geometry (Å, °)

*D*—H⋯*A*	*D*—H	H⋯*A*	*D*⋯*A*	*D*—H⋯*A*
N1—H5⋯O1^i^	0.893 (18)	2.050 (18)	2.9349 (16)	170.8 (16)
N1—H6⋯O3^ii^	0.895 (19)	2.057 (19)	2.9171 (16)	161 (2)
O2—H9⋯O1^iii^	0.909 (19)	1.78 (2)	2.6808 (14)	173 (2)

Symmetry codes: (i) 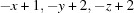; (ii) 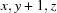; (iii) 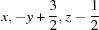.

**Fig. 1.**
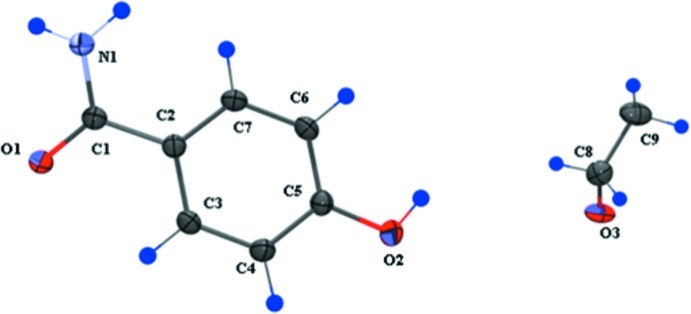
The structure of title the compound with atom labels and 50% probability displacement ellipsoids.

**Fig. 2.**
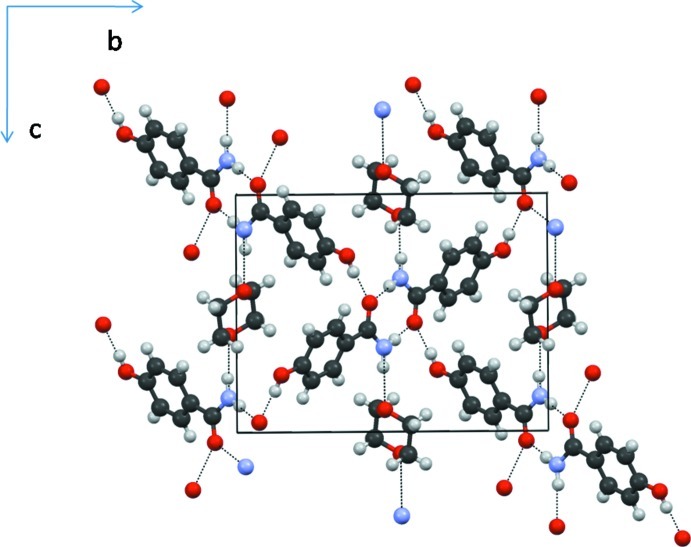
O—H···O, N—H···O supramolecular synthons and amide···amide homodimers in the crystal structure.

## supplementary crystallographic information

### Comment

Hydroxybenzamides and their derivates are extensively used as starting materials in the synthesis of fine chemicals and agrochemicals (Perlovich *et al.*, 2007). Their physicochemical properties are recurrently studied in environmental and biological systems in descriptions of transport and metabolism. The molecular structure of the title compound is shown in Figure 1. In the solvated crystal, molecules are linked by amide···amide homodimers and other O—H···O and N—H···O synthons. The dioxane molecules form a channel along the *a* axis. Their position in the channel is stabilized by N—H···O hydrogen-bonded synthons (Figure 2).

### Experimental

Crystals of the title compound were obtained by slow evaporation of a saturated solution of 4-hydroxybenzamide in 1,4-dioxane at ambient temperature. Good diffraction quality crystals were obtained after five days.

### Refinement

All hydrogen atoms were located from difference Fourier maps and refined isotropically.

### Crystal data

**Table:**

C_7_H_7_NO_2_·0.5C_4_H_8_O_2_	*F*(000) = 384
*M**_r_* = 181.19	*D*_x_ = 1.385 Mg m^−^^3^
Monoclinic, *P*2_1_/*c*	Mo *K*α radiation, λ = 0.71073 Å
Hall symbol: -P 2ybc	Cell parameters from 2599 reflections
*a* = 5.4062 (15) Å	θ = 3.4–27.5°
*b* = 14.530 (3) Å	µ = 0.11 mm^−^^1^
*c* = 12.027 (2) Å	*T* = 150 K
β = 113.117 (10)°	Block, colourless
*V* = 868.9 (3) Å^3^	0.30 × 0.30 × 0.20 mm
*Z* = 4	

### Data collection

**Table:**

Rigaku Mercury375R (2x2 bin mode) diffractometer	1987 independent reflections
Radiation source: fine-focus sealed tube	1841 reflections with *I* > 2σ(*I*)
Graphite monochromator	*R*_int_ = 0.064
profile data from ω–scans	θ_max_ = 27.5°, θ_min_ = 3.4°
Absorption correction: multi-scan (REQAB; Jacobson, 1998)	*h* = −7→7
*T*_min_ = 0.969, *T*_max_ = 0.979	*k* = −18→18
9077 measured reflections	*l* = −15→15

### Refinement

**Table:**

Refinement on *F*^2^	Primary atom site location: structure-invariant direct methods
Least-squares matrix: full	Secondary atom site location: difference Fourier map
*R*[*F*^2^ > 2σ(*F*^2^)] = 0.039	Hydrogen site location: difference Fourier map
*wR*(*F*^2^) = 0.111	All H-atom parameters refined
*S* = 1.01	*w* = 1/[σ^2^(*F*_o_^2^) + (0.059*P*)^2^ + 0.2987*P*] where *P* = (*F*_o_^2^ + 2*F*_c_^2^)/3
1987 reflections	(Δ/σ)_max_ = 0.028
162 parameters	Δρ_max_ = 0.28 e Å^−^^3^
0 restraints	Δρ_min_ = −0.22 e Å^−^^3^

### Special details

**Table:**

Geometry. Bond distances, angles *etc*. have been calculated using the rounded fractional coordinates. All su's are estimated from the variances of the (full) variance-covariance matrix. The cell e.s.d.'s are taken into account in the estimation of distances, angles and torsion angles
Refinement. Refinement on *F*^2^ for ALL reflections except those flagged by the user for potential systematic errors. Weighted *R*-factors *wR* and all goodnesses of fit *S* are based on *F*^2^, conventional *R*-factors *R* are based on *F*, with *F* set to zero for negative *F*^2^. The observed criterion of *F*^2^ > σ(*F*^2^) is used only for calculating -*R*-factor-obs *etc*. and is not relevant to the choice of reflections for refinement. *R*-factors based on *F*^2^ are statistically about twice as large as those based on *F*, and *R*-factors based on ALL data will be even larger.

### Fractional atomic coordinates and isotropic or equivalent isotropic displacement parameters (Å^2^)

**Table:**

	*x*	*y*	*z*	*U*_iso_*/*U*_eq_	
O1	0.80614 (16)	0.92851 (6)	1.04228 (7)	0.0225 (2)	
O2	1.18374 (17)	0.64369 (6)	0.74199 (8)	0.0260 (3)	
N1	0.5317 (2)	0.97339 (7)	0.85611 (9)	0.0239 (3)	
C1	0.7279 (2)	0.92062 (7)	0.93018 (10)	0.0188 (3)	
C2	0.8467 (2)	0.85033 (7)	0.87597 (10)	0.0188 (3)	
C3	0.6951 (2)	0.80863 (8)	0.76593 (10)	0.0223 (3)	
C4	0.8032 (2)	0.73933 (8)	0.71972 (10)	0.0221 (3)	
C5	1.0678 (2)	0.71106 (8)	0.78283 (10)	0.0198 (3)	
C6	1.2219 (2)	0.75283 (8)	0.89296 (10)	0.0233 (3)	
C7	1.1109 (2)	0.82067 (8)	0.93947 (10)	0.0222 (3)	
O3	0.24469 (18)	−0.02513 (7)	0.59392 (8)	0.0324 (3)	
C8	0.0104 (3)	0.04862 (11)	0.40326 (13)	0.0355 (4)	
C9	0.2203 (3)	0.05905 (9)	0.52870 (12)	0.0294 (4)	
H5	0.442 (3)	1.0084 (12)	0.8887 (15)	0.033 (4)*	
H6	0.481 (4)	0.9703 (13)	0.7759 (17)	0.043 (5)*	
H7	1.217 (3)	0.8496 (11)	1.0151 (15)	0.032 (4)*	
H8	1.405 (3)	0.7330 (11)	0.9349 (14)	0.032 (4)*	
H9	1.060 (4)	0.6223 (13)	0.6709 (17)	0.047 (5)*	
H10	0.693 (3)	0.7102 (11)	0.6426 (14)	0.029 (4)*	
H11	0.513 (3)	0.8257 (11)	0.7213 (14)	0.030 (4)*	
H1	−0.014 (4)	0.1061 (14)	0.3610 (17)	0.052 (5)*	
H2	0.071 (4)	−0.0023 (14)	0.3589 (16)	0.044 (5)*	
H3	0.173 (3)	0.1079 (13)	0.5718 (15)	0.039 (4)*	
H4	0.402 (3)	0.0711 (11)	0.5297 (15)	0.033 (4)*	

### Atomic displacement parameters (Å^2^)

**Table:**

	*U*^11^	*U*^22^	*U*^33^	*U*^12^	*U*^13^	*U*^23^
O1	0.0245 (4)	0.0243 (4)	0.0172 (4)	0.0054 (3)	0.0067 (3)	0.0014 (3)
O2	0.0231 (4)	0.0265 (5)	0.0249 (5)	0.0029 (3)	0.0056 (3)	−0.0086 (3)
N1	0.0250 (5)	0.0264 (5)	0.0184 (5)	0.0079 (4)	0.0066 (4)	0.0017 (4)
C1	0.0186 (5)	0.0185 (5)	0.0189 (5)	−0.0008 (4)	0.0071 (4)	0.0014 (4)
C2	0.0201 (5)	0.0179 (5)	0.0187 (5)	0.0002 (4)	0.0078 (4)	0.0010 (4)
C3	0.0180 (5)	0.0255 (6)	0.0197 (5)	0.0020 (4)	0.0035 (4)	0.0002 (4)
C4	0.0207 (5)	0.0242 (6)	0.0182 (5)	−0.0010 (4)	0.0041 (4)	−0.0030 (4)
C5	0.0209 (5)	0.0181 (5)	0.0207 (5)	−0.0009 (4)	0.0085 (4)	−0.0016 (4)
C6	0.0171 (5)	0.0253 (6)	0.0234 (6)	0.0017 (4)	0.0035 (4)	−0.0037 (4)
C7	0.0198 (5)	0.0236 (6)	0.0195 (5)	−0.0004 (4)	0.0038 (4)	−0.0040 (4)
O3	0.0234 (5)	0.0407 (6)	0.0269 (5)	0.0015 (4)	0.0033 (4)	0.0110 (4)
C8	0.0300 (7)	0.0439 (8)	0.0309 (7)	−0.0002 (6)	0.0103 (5)	0.0147 (6)
C9	0.0267 (6)	0.0254 (6)	0.0347 (7)	−0.0032 (5)	0.0106 (5)	−0.0012 (5)

### Geometric parameters (Å, º)

**Table:**

O1—C1	1.2498 (14)	C4—C5	1.3923 (17)
O2—C5	1.3543 (15)	C5—C6	1.3965 (16)
O2—H9	0.909 (19)	C6—C7	1.3814 (17)
O3—C9	1.4309 (17)	C3—H11	0.951 (17)
O3—C8^i^	1.434 (2)	C4—H10	0.980 (16)
N1—C1	1.3284 (16)	C6—H8	0.962 (17)
N1—H5	0.893 (18)	C7—H7	0.961 (17)
N1—H6	0.895 (19)	C8—C9	1.498 (2)
C1—C2	1.4864 (16)	C8—H1	0.96 (2)
C2—C7	1.3973 (17)	C8—H2	1.04 (2)
C2—C3	1.3924 (16)	C9—H3	0.971 (18)
C3—C4	1.3854 (17)	C9—H4	0.993 (18)
			
O1···C5^ii^	3.3571 (17)	C9···H4^xiv^	3.060 (17)
O1···N1^iii^	2.9349 (16)	C9···H6^vi^	3.033 (19)
O1···C1^iv^	3.2567 (17)	H1···C3^xii^	2.98 (2)
O1···O2^ii^	2.6808 (14)	H1···C4^xii^	2.86 (2)
O1···C4^ii^	3.2448 (17)	H2···H3^i^	2.38 (3)
O2···O1^v^	2.6808 (14)	H2···O2^xv^	2.69 (2)
O3···O3^i^	2.8184 (16)	H3···H2^i^	2.38 (3)
O3···N1^vi^	2.9171 (16)	H3···C5^xvi^	2.964 (18)
O1···H7	2.632 (17)	H4···C9^xiv^	3.060 (17)
O1···H5^iii^	2.050 (18)	H4···H8^xvii^	2.54 (2)
O1···H10^ii^	2.544 (16)	H4···H4^xiv^	2.55 (2)
O1···H9^ii^	1.78 (2)	H5···C1^iii^	2.868 (17)
O2···H2^vii^	2.69 (2)	H5···H5^iii^	2.51 (2)
O2···H7^v^	2.806 (17)	H5···O1^iii^	2.050 (18)
O3···H11^vi^	2.721 (16)	H6···C3	2.64 (2)
O3···H6^vi^	2.057 (19)	H6···C9^ix^	3.033 (19)
N1···C8^viii^	3.353 (2)	H6···H11	2.23 (2)
N1···O3^ix^	2.9171 (16)	H6···C8^viii^	2.70 (2)
N1···O1^iii^	2.9349 (16)	H6···O3^ix^	2.057 (19)
N1···H11	2.668 (16)	H7···O1	2.632 (17)
C1···O1^iv^	3.2567 (17)	H7···O2^ii^	2.806 (17)
C1···C1^iv^	3.5941 (19)	H7···H10^xviii^	2.58 (2)
C1···C6^x^	3.5589 (19)	H7···H9^ii^	2.38 (3)
C3···C6^x^	3.5503 (19)	H8···H4^xix^	2.54 (2)
C4···O1^v^	3.2448 (17)	H8···H10^xviii^	2.51 (2)
C5···O1^v^	3.3571 (17)	H9···O1^v^	1.78 (2)
C6···C1^xi^	3.5589 (19)	H9···H10	2.27 (3)
C6···C3^xi^	3.5503 (19)	H9···H7^v^	2.38 (3)
C8···N1^viii^	3.353 (2)	H9···C1^v^	2.813 (19)
C1···H5^iii^	2.868 (17)	H9···C7^v^	3.02 (2)
C1···H9^ii^	2.813 (19)	H10···H7^xx^	2.58 (2)
C3···H1^xii^	2.98 (2)	H10···H8^xx^	2.51 (2)
C3···H6	2.64 (2)	H10···H9	2.27 (3)
C4···H1^xii^	2.86 (2)	H10···O1^v^	2.544 (16)
C5···H3^xiii^	2.964 (18)	H11···H6	2.23 (2)
C7···H9^ii^	3.02 (2)	H11···O3^ix^	2.721 (16)
C8···H6^viii^	2.70 (2)	H11···N1	2.668 (16)
			
C5—O2—H9	108.3 (14)	C4—C3—H11	118.0 (10)
C8^i^—O3—C9	109.51 (11)	C5—C4—H10	120.1 (10)
H5—N1—H6	120.9 (17)	C3—C4—H10	119.9 (10)
C1—N1—H5	117.6 (11)	C5—C6—H8	118.4 (9)
C1—N1—H6	121.3 (14)	C7—C6—H8	121.5 (9)
N1—C1—C2	118.11 (10)	C2—C7—H7	119.0 (10)
O1—C1—N1	120.89 (10)	C6—C7—H7	120.2 (10)
O1—C1—C2	120.98 (10)	O3^i^—C8—C9	110.84 (12)
C1—C2—C7	119.81 (10)	O3—C9—C8	109.64 (12)
C3—C2—C7	118.63 (10)	C9—C8—H1	109.7 (12)
C1—C2—C3	121.43 (10)	C9—C8—H2	108.8 (11)
C2—C3—C4	120.93 (11)	H1—C8—H2	110.7 (16)
C3—C4—C5	120.04 (10)	O3^i^—C8—H1	106.6 (14)
O2—C5—C4	122.57 (10)	O3^i^—C8—H2	110.1 (12)
C4—C5—C6	119.44 (11)	O3—C9—H3	108.6 (10)
O2—C5—C6	118.00 (10)	O3—C9—H4	105.4 (9)
C5—C6—C7	120.13 (11)	C8—C9—H3	111.0 (10)
C2—C7—C6	120.80 (10)	C8—C9—H4	112.6 (10)
C2—C3—H11	121.1 (10)	H3—C9—H4	109.4 (14)
			
C9—O3—C8^i^—C9^i^	58.92 (14)	C3—C2—C7—C6	1.44 (17)
C8^i^—O3—C9—C8	−58.20 (15)	C2—C3—C4—C5	−0.65 (18)
N1—C1—C2—C3	−30.42 (16)	C3—C4—C5—C6	0.29 (17)
N1—C1—C2—C7	153.69 (11)	C3—C4—C5—O2	180.00 (13)
O1—C1—C2—C7	−27.88 (16)	O2—C5—C6—C7	−178.77 (11)
O1—C1—C2—C3	148.02 (11)	C4—C5—C6—C7	0.93 (17)
C1—C2—C7—C6	177.45 (10)	C5—C6—C7—C2	−1.81 (18)
C1—C2—C3—C4	−176.15 (11)	O3^i^—C8—C9—O3	58.99 (16)
C7—C2—C3—C4	−0.21 (17)		

Symmetry codes: (i) −*x*, −*y*, −*z*+1; (ii) *x*, −*y*+3/2, *z*+1/2; (iii) −*x*+1, −*y*+2, −*z*+2; (iv) −*x*+2, −*y*+2, −*z*+2; (v) *x*, −*y*+3/2, *z*−1/2; (vi) *x*, *y*−1, *z*; (vii) *x*+1, −*y*+1/2, *z*+1/2; (viii) −*x*, −*y*+1, −*z*+1; (ix) *x*, *y*+1, *z*; (x) *x*−1, *y*, *z*; (xi) *x*+1, *y*, *z*; (xii) −*x*+1, −*y*+1, −*z*+1; (xiii) −*x*+1, *y*+1/2, −*z*+3/2; (xiv) −*x*+1, −*y*, −*z*+1; (xv) *x*−1, −*y*+1/2, *z*−1/2; (xvi) −*x*+1, *y*−1/2, −*z*+3/2; (xvii) −*x*+2, *y*−1/2, −*z*+3/2; (xviii) *x*+1, −*y*+3/2, *z*+1/2; (xix) −*x*+2, *y*+1/2, −*z*+3/2; (xx) *x*−1, −*y*+3/2, *z*−1/2.

### Hydrogen-bond geometry (Å, º)

**Table:**

*D*—H···*A*	*D*—H	H···*A*	*D*···*A*	*D*—H···*A*
N1—H5···O1^iii^	0.893 (18)	2.050 (18)	2.9349 (16)	170.8 (16)
N1—H6···O3^ix^	0.895 (19)	2.057 (19)	2.9171 (16)	161 (2)
O2—H9···O1^v^	0.909 (19)	1.78 (2)	2.6808 (14)	173 (2)

Symmetry codes: (iii) −*x*+1, −*y*+2, −*z*+2; (v) *x*, −*y*+3/2, *z*−1/2; (ix) *x*, *y*+1, *z*.
